# Supplementary material for: MELK expression correlates with tumor mitotic activity but is not required for cancer growth
Source: eLife. 2018 Feb 8;7:e32838. doi: 10.7554/eLife.32838 (PMC5805410; doi:10.7554/eLife.32838)
Supplement: Transparent reporting form [file elife-32838-transrepform.docx]

***eLife’s* transparent reporting form**

We encourage authors to provide detailed information *within their submission* to facilitate the interpretation and replication of experiments. Authors can upload supporting documentation to indicate the use of appropriate reporting guidelines for health-related research (see [EQUATOR Network](http://www.equator-network.org/%20)), life science research (see the [BioSharing Information Resource](https://biosharing.org/)), or the [ARRIVE guidelines](http://www.plosbiology.org/article/info:doi/10.1371/journal.pbio.1000412) for reporting work involving animal research. Where applicable, authors should refer to any relevant reporting standards documents in this form.

If you have any questions, please consult our Journal Policies and/or contact us: [editorial@elifesciences.org](mailto:editorial@elifesciences.org).

**Sample-size estimation**

- You should state whether an appropriate sample size was computed when the study was being designed
- You should state the statistical method of sample size computation and any required assumptions
- If no explicit power analysis was used, you should describe how you decided what sample (replicate) size (number) to use

Sample sizes were not pre-determined.

Please outline where this information can be found within the submission (e.g., sections or figure legends), or explain why this information doesn’t apply to your submission:

**Replicates**

- You should report how often each experiment was performed
- You should include a definition of biological versus technical replication
- The data obtained should be provided and sufficient information should be provided to indicate the number of independent biological and/or technical replicates
- If you encountered any outliers, you should describe how these were handled
- Criteria for exclusion/inclusion of data should be clearly stated
- High-throughput sequence data should be uploaded before submission, with a private link for reviewers provided (these are available from both GEO and ArrayExpress)

Please outline where this information can be found within the submission (e.g., sections or figure legends), or explain why this information doesn’t apply to your submission:

Figure 1: Soft agar assays were performed at least twice for each cell line with the mouse and human MELK constructs.

Figure 2A: Dilution plate assays were completed once for each cell line.

Figure 2B-C: Soft agar assays were completed once for each cell line.

Figure 2D-E: Mammosphere growth assays were performed twice for each cell line.

Figure 2F-D: Xenograft injections were performed on 6 to 10 mice each, as indicated in the figure.

Figure 3: Drug sensitivity and growth in stressful conditions assays were performed once for each cell line.

Figure 4A: HTH-01-091 treatment demonstrating the degradation of MELK was performed at least twice for each cell line.

Figure 4B-C: Soft agar assays and drug treatment assays were performed once for each cell line.

Supplemental Figure 1: Westerns on mitotically arrested cells were performed once for each cell line.

Supplemental Figure 2A: TOPO-sequencing was performed on 20 colonies from A375 cell lines and 10 colonies from DLD1 cell lines.

Supplemental Figure 2B: Westerns demonstrating complete MELK knockout were performed at least twice.

Supplemental Figure 2C-D: Proliferation assays and drug sensitivity assays were performed once for each cell line.

Supplemental Figure 3A: Westerns showing ablation of MELK protein in heterogeneous populations were completed at least twice.

Supplemental Figure 3B: Xenograft injections were performed in the number of mice indicated in the figure.

Supplemental Figure 4: Westerns shown were performed at least twice.

**Statistical reporting**

- Statistical analysis methods should be described and justified
- Raw data should be presented in figures whenever informative to do so (typically when N per group is less than 10)
- For each experiment, you should identify the statistical tests used, exact values of N, definitions of center, methods of multiple test correction, and dispersion and precision measures (e.g., mean, median, SD, SEM, confidence intervals; and, for the major substantive results, a measure of effect size (e.g., Pearson's r, Cohen's d)
- Report exact p-values wherever possible alongside the summary statistics and 95% confidence intervals. These should be reported for all key questions and not only when the p-value is less than 0.05.

Please outline where this information can be found within the submission (e.g., sections or figure legends), or explain why this information doesn’t apply to your submission:

Visual inspection of most data revealed overlap between the results obtained with control and MELK-knockout cell lines. Thus, for most of the data in this paper, no statistical methods were used to compare the samples, as a non-significant result was demonstrated by the overlap in data.

(For large datasets, or papers with a very large number of statistical tests, you may upload a single table file with tests, Ns, etc., with reference to sections in the manuscript.)

**Group allocation**

- Indicate how samples were allocated into experimental groups (in the case of clinical studies, please specify allocation to treatment method); if randomization was used, please also state if restricted randomization was applied
- Indicate if masking was used during group allocation, data collection and/or data analysis

Please outline where this information can be found within the submission (e.g., sections or figure legends), or explain why this information doesn’t apply to your submission:

N/A

**Additional data files (“source data”)**

- We encourage you to upload relevant additional data files, such as numerical data that are represented as a graph in a figure, or as a summary table
- Where provided, these should be in the most useful format, and they can be uploaded as “Source data” files linked to a main figure or table
- Include model definition files including the full list of parameters used
- Include code used for data analysis (e.g., R, MatLab)
- Avoid stating that data files are “available upon request”

Please indicate the figures or tables for which source data files have been provided:

Source code for data presented in Figure 5 can be found here: <https://github.com/joan-smith/survival-analysis-scripts>
